# Editorial: Visuospatial and visuoconstructional abilities and disorders across the life span

**DOI:** 10.3389/fpsyt.2025.1604144

**Published:** 2025-04-28

**Authors:** Isa Zappullo, Massimiliano Conson, Petra Jansen, Irene C. Mammarella, Luigi Trojano

**Affiliations:** ^1^ Developmental Neuropsychology Laboratory, Department of Psychology, University of Campania Luigi Vanvitelli, Caserta, Italy; ^2^ Faculty of Human Sciences, University of Regensburg, Regensburg, Germany; ^3^ Department of Developmental and Social Psychology, University of Padua, Padua, Italy; ^4^ Neuropsychology Laboratory, Department of Psychology, University of Campania Luigi Vanvitelli, Caserta, Italy

**Keywords:** visuospatial, visual agnosia, unilateral spatial neglect, mental rotation, perspective taking, navigation

Visuospatial cognition covers a wide range of non-verbal cognitive abilities that are pivotal for enabling individuals to interact with the environment effectively, and are strongly linked to drawing and assembling (visuoconstructional) activities. This link is evident both in childhood, when the progressive maturation of visuospatial cognition parallels changes in performance on spatial construction tasks (1, 2), and in adulthood, when acquired visuospatial deficits and disorders of spatial construction are frequently observed together following brain damage (3–5). Visuospatial skills are affected by a large variety of factors, spanning from demographics (e.g., sex, age, and education) to individual (e.g., personality, autistic traits, and spatial self-evaluations) and cultural (e.g., gender stereotypes) differences (6–9).

The present Research Topic offers the latest evidence on visuospatial and visuoconstructional abilities and disorders across the lifespan, encompassing studies on typically and atypically developing children (Ebert et al.; Orefice et al.), healthy adults (Ricci et al.; Muffato et al.), and individuals with acquired brain lesions (Lederer et al.; Moretta et al.).

Ebert et al. investigated the link between implicit and explicit spatial gender stereotypes and mental rotation performance in preschool-aged typically developing children. Children displayed explicit stereotypes in favor of boys, stronger in boys than girls. Evidence of implicit stereotypes favoring boys was also found in the overall sample, but independently from sex. Although no clear relationships were found between stereotypes and mental rotation, a positive association emerged in girls between implicit stereotypes favoring their own sex and mental rotation. These findings support the existence of explicit spatial gender stereotypes as early as at pre-school age.

Orefice et al. investigated several aspects of visuospatial cognition in children with Developmental Visuospatial Disorder (DVSD) and Developmental Coordination Disorder (DCD). Compared with typical controls, both groups showed significantly lower performance on a visuospatial perspective-taking task (VSPT), with the DVSD group scoring the lowest. Interestingly, group moderated the relationship between VSPT and fine-motor coordination, showing an opposite pattern between the clinical groups (a positive association for the DVSD group and the reverse pattern in the DCD group). These findings suggest that children with DVSD tend to use a strategy relying on fine-motor skills when performing VSPT task, whereas DCD children would not. A strict link between visuomotor coordination and other high-order visuospatial abilities has also been reported in typical development (10, 11).

Ricci et al. assessed the interaction between visuospatial attention and action control in healthy adult individuals. The authors tested the effect of the hand starting position (near or far from the body) on the subjective midpoint’s localization in a radial line bisection task. Results showed that participants bisected radial lines farther than the true center in both near and far conditions, although bisection errors were greater in the near condition. These findings provide new evidence for a classical model postulating close relationships between attention and action (12).

Moreover, Muffato et al. examined the ability to navigate combining scales of sense of direction and spatial representation, spatial anxiety and attitude in exploring across the adult lifespan. A distinct age-related pattern in the different self-evaluations emerged, with a linear positive trend for a sense of direction, and an age breakpoint (66 and 71 years) for the other two tasks, with an increase in spatial anxiety and a decrease in attitude towards exploring. Moreover, men produced higher self-ratings than women for sense of direction and attitude towards exploring (and a lower spatial anxiety); higher education was associated with lower spatial anxiety and higher sense of direction. These results contribute to shed light on individual differences in navigational skills across the adult lifespan, thus further enlarging the range of issues relevant to visuospatial cognition.

Finally, two studies assessed visuospatial cognition in adult patients with brain lesions. Lederer et al. investigated the kinematics of grasping toward common objects in an adult with acquired visual agnosia who showed considerable residual everyday skills. The patient was able to recognize most colored objects with a variable delay, but he was strongly impaired in recognizing objects in a color-masked condition, and his kinematic grasping performance was significantly slower than that of matched healthy controls, both when he was instructed to grasp-then-name and to name-then-grasp the target objects. These findings suggest that color information can be processed by the dorsal stream and contribute to visuomotor transformations (13).

In a systematic review, Moretta et al. examined a well-known disorder of spatial cognition, i.e., unilateral spatial neglect, but focusing on a peculiar manifestation implying a defective exploration of the vertical dimension of space. Analysis of the available literature revealed that vertical neglect is more common in lower than upper space, is often associated with attentional bias in the horizontal dimension, and can co-occur with radial space impairment, particularly in the near radial. Vertical neglect is most often assessed by paper-and-pencil tasks, such as line bisection, but computer-based tasks are increasingly employed. Furthermore, the authors also found that the most frequent etiology is vascular, especially ischaemic, involving diverse subcortical and cortical areas, although the temporal lobe is most often affected.

The heterogeneous range of abilities and disorders included within the broad domain of spatial cognition and the variety of methods featured in the present Research Topic highlighted, on the one hand, the complexity of this field of investigation and, on the other hand, delineated the potential for establishing a shared framework. This common ground could help integrate approaches that have largely evolved along separate lines of research.
